# Characteristics of the bacterial microbiome in association with common intestinal parasites in irritable bowel syndrome

**DOI:** 10.1038/s41424-018-0027-2

**Published:** 2018-06-19

**Authors:** Laura Rindom Krogsgaard, Lee O ‘Brien Andersen, Thor Bech Johannesen, Anne Line Engsbro, Christen Rune Stensvold, Henrik Vedel Nielsen, Peter Bytzer

**Affiliations:** 1 Department of Gastroenterology, Zealand University Hospital, Lykkebækvej 1, DK–4600 Køge, Denmark; 2 Department of Clinical Medicine, University of Copenhagen, København, Denmark; 3 Department of Bacteria, Parasites & Fungi, Statens Serum Institut, Artillerivej 5, DK–2300 Copenhagen S, Denmark; 4 Department of Clinical Microbiology, Copenhagen University Hospital, Kettegård Alle 30, DK–2560 Hvidovre, Denmark

## Abstract

**Objective:**

A low prevalence of intestinal parasites has been identified in individuals with irritable bowel syndrome (IBS), but potential associations with alterations in the bacterial microbiome remain largely unexplored. We aimed to investigate the relationship between parasites and bacteria in individuals with IBS in order to identify potential trans-kingdom microbial characteristics.

**Design:**

Stool samples were collected from the Danish background population classified into IBS (*n* = 119), unspecific gastrointestinal (GI) symptoms (*n* = 114), and asymptomatic controls (*n* = 186) based on the Rome III criteria for IBS. Bacterial (16S) and eukaryotic (18S) ribosomal DNA was sequenced, and 18S data were merged with data from conventional parasite laboratory tests. The bacterial microbiome was analyzed according to symptom group and parasite colonization status.

**Results:**

Bacterial richness and diversity were similar for IBS and controls but higher in those with unspecific GI symptoms. A higher abundance of Bacteroides and a lower abundance of Faecalibacterium were detected in individuals with IBS and unspecific GI symptoms compared with controls. Principal component analyses indicated differences in bacterial composition related to parasite colonization rather than symptom group. Parasites were detected at the lowest frequency in the IBS group (39%) and in samples dominated by Bacteroides. Higher bacterial richness and diversity were found in parasite-positive samples from controls and those with unspecific GI symptoms but not in individuals with IBS.

**Conclusion:**

Parasite colonization, rather than bacterial composition, differed between individuals with IBS and healthy controls. Parasite colonization was associated to a rich and diverse bacterial microbiome; however, this association was altered in IBS.

## Introduction

The intestinal parasites *Dientamoeba fragilis* and *Blastocystis* were previously found to colonize individuals with IBS at a lower frequency compared with asymptomatic controls^1^. This finding indicates that our understanding of the role of some intestinal parasites in gastrointestinal (GI) health and disease is limited. Several studies have reported a difference in the composition of the bacterial microbiome in relation to IBS^2–16^, although with inconsistent findings. Organisms from the different kingdoms of the gut microbiome coexist and interact^17^, and given the fact that some intestinal parasites, such as *Blastocystis* and *D. fragilis*, are common colonizers of the human GI tract^1,18,19^, it is relevant to investigate the lower prevalence of intestinal parasites in individuals with IBS in relation to possible differences in the bacterial microbiome. Limited investigation in the field has focused on the association between *Blastocystis* and the bacterial microbiome in IBS^11,20^, identifying differences across some bacterial groups in relation to colonization with *Blastocystis*.

The association between parasites, including *D. fragilis* and *Blastocystis*, and the bacterial microbiome in IBS is still largely unexplored, but could contribute to a trans-kingdom understanding of the possible role of the microbiome in IBS.

The aim of this study was to investigate characteristics of the bacterial microbiome in IBS related to colonization with intestinal parasites, focusing on *D. fragilis* and *Blastocystis*, by comparing stool microbiome data from individuals with IBS with asymptomatic controls and with individuals with GI symptoms not fulfilling the criteria for IBS.

## Methods

We conducted an internet-based cohort study of the Danish background population in 2010, with a follow-up in 2011; described elsewhere^1,21^. Briefly, the cohort consisted of 6112 persons (age, 18–50 years [yr]), who completed a questionnaire based on the Rome III criteria for IBS^22^. Respondents were classified into four symptom groups: asymptomatic controls, individuals with IBS (fulfilling the Rome III criteria for IBS and reporting no organic GI diagnosis) subtyped by the Rome III criteria according to stool type^22^, individuals with unspecific GI symptoms (reporting GI symptoms within the past 3 months not fulfilling the Rome III criteria for IBS), and individuals with organic GI disease (reporting a GI diagnosis).

The classification into symptom groups by the web-based questionnaire was validated by telephone interviews conducted by a blinded physician on a sample of the population as described elsewhere^21^.

Consecutive respondents were asked to provide stool samples, until a pre-specified number was reached (see the paragraph on Statistics for details). Sampling kits were sent by postal mail to those agreeing to submit stool samples. The kit included two test tubes without additives. Samples were returned by mail, delivered in the laboratory the following day. Upon receipt, stool samples were tested for ova and parasites by microscopy, short-term in vitro culture for *Blastocystis*^23^, and a multiplex quantitative polymerase chain reaction (qPCR) for *D. fragilis, Cryptosporidium* spp., *Entamoeba histolytica*, *Entamoeba dispar*, and *Giardia intestinalis*^24–26^. Purified DNA was amplified using four primers targeting nuclear ribosomal genes (16S for prokaryotes and 18S for eukaryotes). For prokaryotes, a modified version of the published universal prokaryotic primers 341F/806R^27^ was used, while three in-house primers (G3F1/G3R1, G4F3/G4R3, and G6F1/G6R1) were used to amplify eukaryotic DNA (see supplementary material for details). Sequences were mapped using BION, a k-mer-based mapping software described previously^28^. A unique taxon was defined by denotation to a specific evolutionary taxonomic group.

The data on fungi are still undergoing taxonomic validation and will be presented elsewhere. Parasite sequencing data were merged with data from routine laboratory tests, including microscopy, culture, and qPCR presented earlier^1^ to ensure the broadest possible and most sensitive approach to parasite detection. Four categories of parasite colonization status were studied in relation to the composition of bacteria: (i) any parasite-positive/-negative, (ii) *D. fragilis*-positive/-negative, (iii) *Blastocystis*-positive/-negative, and (iv) positive for multiple parasites (>1)/not positive for multiple parasites (≤1).

### Statistics

Sample size was estimated with the aim to detect a statistical difference at a significance level of 5% in the prevalence of intestinal parasites between individuals with IBS (expected prevalence, 30%^29^) and asymptomatic controls (expected prevalence, 12%^30^). Seventy-three persons with IBS and 73 controls were required, and we aimed to receive samples from 100 individuals in each group. In 2010, we requested stool samples from 200 individuals with IBS and 300 controls. In 2011, we requested samples from 200 individuals with unspecific GI symptoms.

Differences in the proportion of positive samples according to the four parasite colonization categories between the symptom groups were tested by the *χ*^2^-test. BION data were analyzed in R^31^ with the PhyloSeq^32^, and Vegan packages^33^, using ggplot2^34^ and Plotly^35^ for graphical presentation.

Data were rarefied to 10,000 sequences in order to enable comparison of data from samples with a large variation in number of sequence reads. Richness was defined as a simple count of unique taxa and reported as the mean (standard deviation [SD]) of observed unique taxa. Shannon’s diversity index was used as a measure of diversity, defined by two components, richness and evenness; the latter is a term referring to how equal the abundance of unique taxa is. Differences in bacterial richness and diversity were compared according to symptom group and according to parasite colonization status by two-sided *t*-tests (when comparing two groups) or one-way ANOVA (when comparing more than two groups). In order to visualize general differences of the bacterial microbiome according to symptom group and parasite colonization status, principal coordinate analyses (PCoA), which reflects inter-sample dissimilarity, were carried out based on Bray–Curtis dissimilarity of the rarefied data. Relative abundance data were based on rarefied sequences and refers to the percentage of total sequences in a sample assigned to a particular taxon. The relative abundance of the two most common phyla and genera was compared between symptom groups by a two-sided *t-*test, and bar plots were used to display the relative abundance of the ten most common genera for each symptom group.

Based on presence/absence of species, we hypothesized that if any bacterial species should have a significant role in IBS, it should be present at a lower or higher prevalence in the IBS group. We defined that potential species of relevance to IBS should be present with a difference in prevalence of at least 10% between the IBS group and asymptomatic controls based on the number of samples collected. Differences in prevalence of the selected species between the IBS group and asymptomatic controls were tested for statistical significance by the *χ*^2^-test.

Species present at a higher or lower prevalence of at least 10% (1) in the group with unspecific GI symptoms compared with the IBS group and/or the control group and (2) between positive and negative samples across the four parasite colonization status categories were also reported.

All authors had access to the study data and reviewed and approved the final manuscript. The study was approved by the ethics committee in Region Zealand, Denmark (SJ-144 and SJ-199) and by the Danish Data Protection Agency. The study was performed according to the Declaration of Helsinki.

## Results

Stool samples were obtained from 124 individuals with IBS and 204 asymptomatic controls in 2010 and from 118 individuals with unspecific GI symptoms in 2011. We performed 16S rRNA and 18S rRNA gene sequencing on 119 samples from individuals with IBS (62% women; mean age, 37.2 yr [SD: 7.8]), 186 samples from asymptomatic controls (39% women; mean age, 37.8 yr [SD: 8.1]), and 114 persons with unspecific GI symptoms (68% women; mean age, 36.4 yr [SD: 8.0]). For the remaining samples, stool was not stored for DNA extraction by mistake. IBS subtypes according to stool pattern were distributed as follows: Mixed-IBS, 49 (41%); IBS with diarrhea, 38 (32%); IBS with constipation, 19 (16%); and un-subtyped IBS, 10 (8%) (missing data for three individuals).

### Parasite colonization

Of the 419 samples analyzed, 197 (47%) samples were positive for parasites with 67 (16%) samples being positive for multiple parasite species. There was no difference in frequency of colonization with parasites according to sex or age (data not shown). Parasite colonization status for all three symptom groups is presented in Table 1. The prevalence of parasites was highest in asymptomatic individuals and lowest in the IBS group across all four parasite colonization categories.

**Table 1:** Prevalence of intestinal parasites in the three symptom groups

	Positive for any parasite, *n* (%)	*Dientamoeba fragilis*-positive, *n* (%)	*Blastocystis-*positive, *n* (%)	Positive for multiple parasites species, *n* (%)
Individuals with IBS, *n* = 119	46 (39)	29 (24)	21 (18)	12 (10)
Individuals with unspecific GI symptoms, *n* = 114	54 (48)	34 (30)	28 (25)	17 (15)
Asymptomatic controls, *n* = 186	97 (52)	71 (38)	51 (27)	38 (20)
*P* value IBS vs asymptomatic	0.02	0.01	0.05	0.02

Difference in prevalence tested by *χ*^2^-test

*GI* gastrointestinal, *IBS* irritable bowel syndrome

An overview of parasites detected by sequencing and routine laboratory methods are listed at genus level in Table S1. The primers used to amplify eukaryotic DNA appeared to have low sensitivity with regard to detection of *D. fragilis* and to some extent for *Entamoeba* spp.

### Bacterial composition

#### Richness and diversity

BION mapped 38,534,562 sequences to 1536 unique taxa across all 419 samples. Richness and diversity did not differ between the IBS and the control group; however, higher diversity and richness were found in samples from individuals with unspecific GI symptoms (Table 2). Richness and diversity did not differ according to IBS subtype or time since onset of symptoms (data not shown). Richness and diversity were significantly higher in samples positive for any parasite, D. fragilis or Blastocystis, and multiple parasite species (Table 2). For asymptomatic individuals and those with unspecific GI symptoms, bacterial richness and diversity was higher in parasite-positive samples in the four parasite colonization status categories (Table 3). However, in the IBS group, richness did not differ according to parasite status, and diversity was only higher in samples positive for Blastocystis or multiple parasites (Table 3).

**Table 2:** Bacterial richness and diversity according to symptom group and parasite colonization status

	Observed unique taxa Mean (SD) [*P* value]	Shannon’s diversity index Mean (SD) [*P* value]
Individuals with IBS, *n* = 119	117.6 (16.6) [0.36]^a^	3.00 (0.29) [0.59]^a^
Asymptomatic controls, *n* = 186	115.8 (16.6)	2.98 (0.32)
Individuals with unspecific GI symptoms, *n* = 114	126.0 (21.0) [<0.0001]^b^	3.07 (0.31) [0.06]^b^
Positive for any parasite, *n* = 197	124.2 (17.5) [<0.0001]^c^	3.09 (0.3) [<0.0001]^c^
Negative for any parasite, *n* = 222	114.5 (17.9)	2.94 (0.3)
*Dientamoeba fragilis*-positive, *n* = 134	124.0 (17.0) [<0.0001]^c^	3.08 (0.3) [0.002]^c^
*Dientamoeba fragilis*-negative, *n* = 285	116.7 (18.6)	2.98 (0.3)
*Blastocystis*-positive, *n* = 100	126.0 (18.1) [<0.0001]^c^	3.11 (0.2) [<0.0001]^c^
*Blastocystis*-negative, *n* = 319	116.9 (17.9)	2.98 (0.3)
Positive for multiple parasite species, *n* = 67	126.3 (16.0) [<0.001]^c^	3.11 (0.3) [<0.001]^c^
Negative for multiple parasite species, *n* = 352	117.7 (18.5)	2.99 (0.3)

*GI* gastrointestinal, *IBS* irritable bowel syndrome, *SD* standard deviation

^a^Difference between the IBS group and asymptomatic control group tested by two-sided *t*-test

^b^Difference between all three symptom groups tested by one-way ANOVA

^c^Difference between positive and negative samples tested by two-sided *t*-test

**Table 3:** Bacterial diversity and richness according to parasite colonization status, specified for each symptom group

Mean (SD)	Positive for any parasite	Negative for any parasite	*Dientamoeba fragilis*-positive	*Dientamoeba fragilis*-negative	*Blastocystis*-positive	*Blastocystis*-negative	Positive for multiple parasite species	Negative for multiple parasite species
IBS, *n* = 119								
Observed unique taxa	120.6 (14.2)	115.7 (17.7)	121.6 (12.5)	116.3 (17.6)	121.3 (16.5)	116.8, 16.6	123.2 (13.3)	117.0 (16.8)
Shannon’s diversity index	3.03 (0.3)	2.98 (0.3)	3.00 (0.3)	3.00 (0.3)	3.12 (0.2)^a^	2.98 (0.3)	3.19 (0.2)^a^	2.98 (0.3)
Unspecific GI symptoms, *n* = 114								
Observed unique taxa	132.9 (21.2)^b^	119.7 (19.0)	133.4 (22.3)^a^	122.8 (19.8)	136.4 (19.0)^a^	122. 6 (20.6)	137.5 (14.9)^a^	124.0 (21.4)
Shannon’s diversity index	3.18 (0.3)^b^	2.97 (0.3)	3.16 (0.3)^a^	3.03 (0.3)	3.21 (0.1)^b^	3.02 (0.3)	3.22 (0.2)^a^	3.04 (0.3)
Asymptomatic, *n* = 186								
Observed unique taxa	121.1 (15.1)^a^	110. (16.3)	120.5 (14.0)^b^	112.9 (17.5)	122.2 (16.0)^b^	113.4 (16.2)	122.2 (15.2)^a^	114.2 (16.6)
Shannon’s diversity index	3.08 (0.3)^b^	2.88 (0.3)	3.07 (0.3)^a^	2.93 (0.3)	3.06 (0.3)^a^	2.96 (0.3)	3.04 (0.3)	2.96 (0.3)

Difference between groups tested by a two-sided *t*-test

*GI* gastrointestinal, *IBS* irritable bowel syndrome, *SD* standard deviation

^a^Difference between positive and negative samples significant with a *P* value ≤0.05

^b^Difference between positive and negative samples significant with a *P* value ≤0.0001

### PCoA

**Fig. 1:**
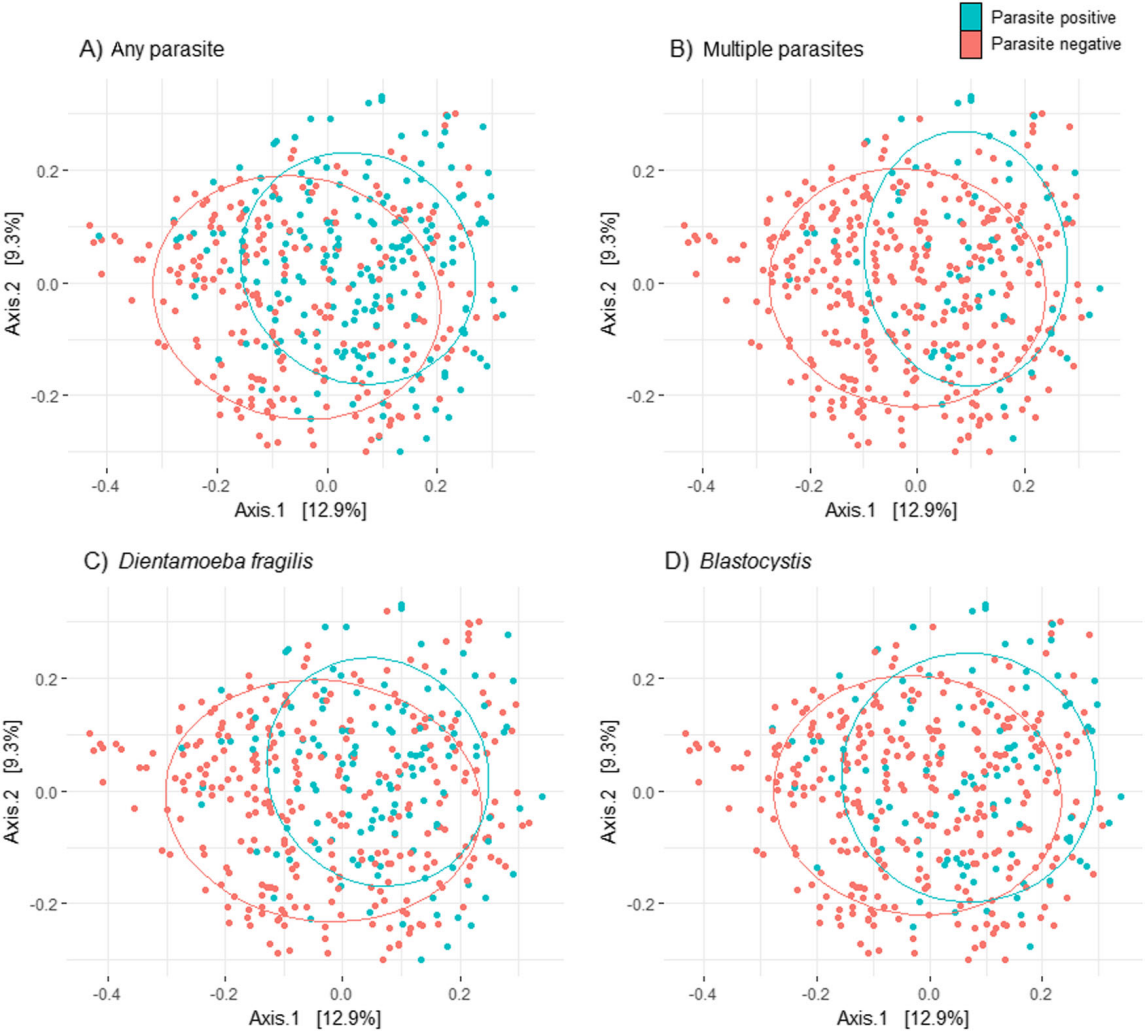
PCoA of the bacterial microbiome according to parasite colonisation status. Principal coordinate analysis visualizing differences in the bacterial microbiome between samples positive and negative for any parasite (**a**), multiple parasites (**b**), Dientamoeba fragilis (**c**), and Blastocystis (**d**). All samples are included in the analysis (*n* = 419). Blue dots represent samples positive in the given parasite category while negative samples are represented by red dots. The closer the dots are ordinated, the more similar the microbiome. Axes summarize the variability in the data, and the value indicates the variation captured in the axis

PCoA revealed no separation of samples according to symptom group, indicating no clear differences in the bacterial microbiome composition between individuals with IBS, individuals with unspecific GI symptoms, and asymptomatic controls (Figure S1). According to parasite colonization status, there was no clear separation of samples; nevertheless, a relative clustering of parasite-positive samples was apparent, indicating that the presence of parasites is associated with basic differences in the bacterial microbiome (Fig. 1).

### Relative abundance

**Fig. 2:**
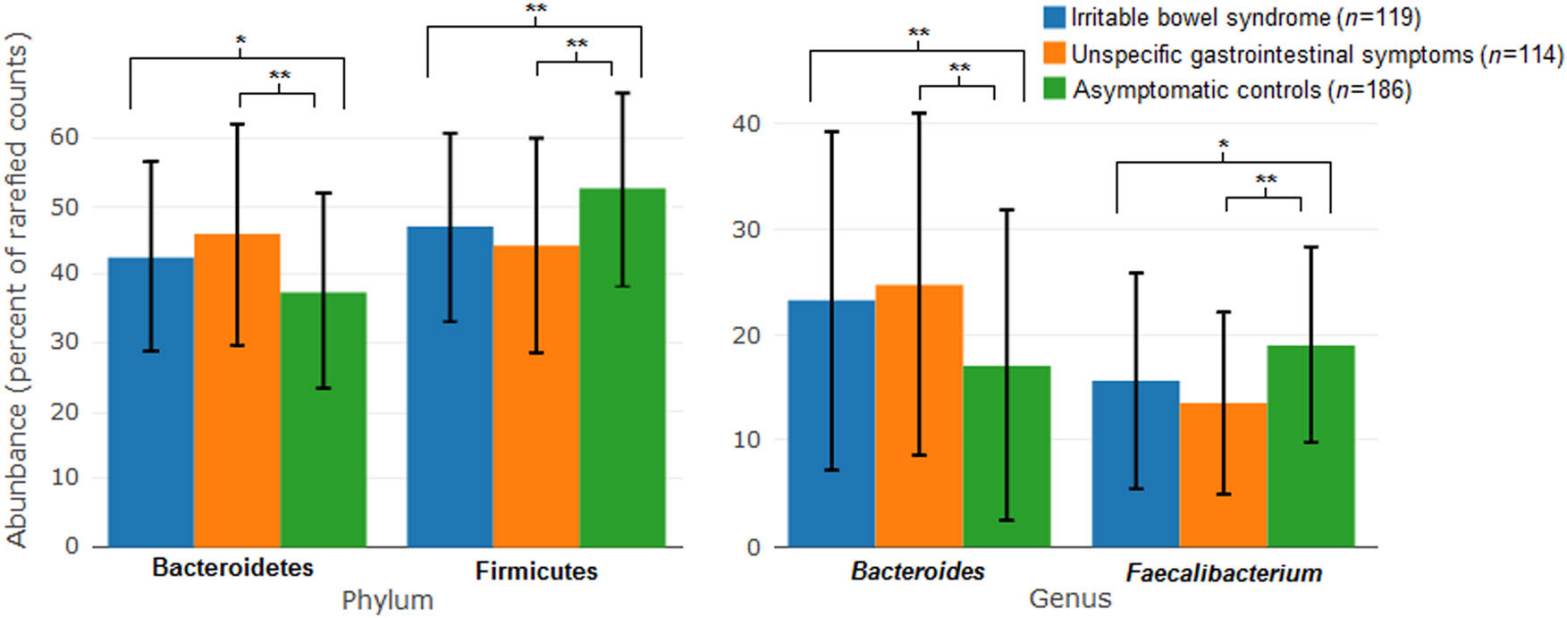
Mean relative abundance of the two most abundant phyla and genera in the three symptom groups. Error bars indicate 1 s.d. of the mean. A significant difference between symptom groups according to a two-sided *t*-test is marked with *p < 0.05 or **p < 0.001

The IBS group had a higher abundance of *Bacteroidetes* and a lower abundance of *Firmicutes* compared with asymptomatic controls (Fig. 2). This was reflected in a higher abundance of *Bacteroides* and a lower abundance of *Faecalibacterium* at genus level in the IBS group compared with asymptomatic controls (Fig. 2). There was a similar difference in the dominating phyla and genera between the group with unspecific GI symptoms and the asymptomatic controls, and with even more pronounced differences between the groups.

**Fig. 3:**
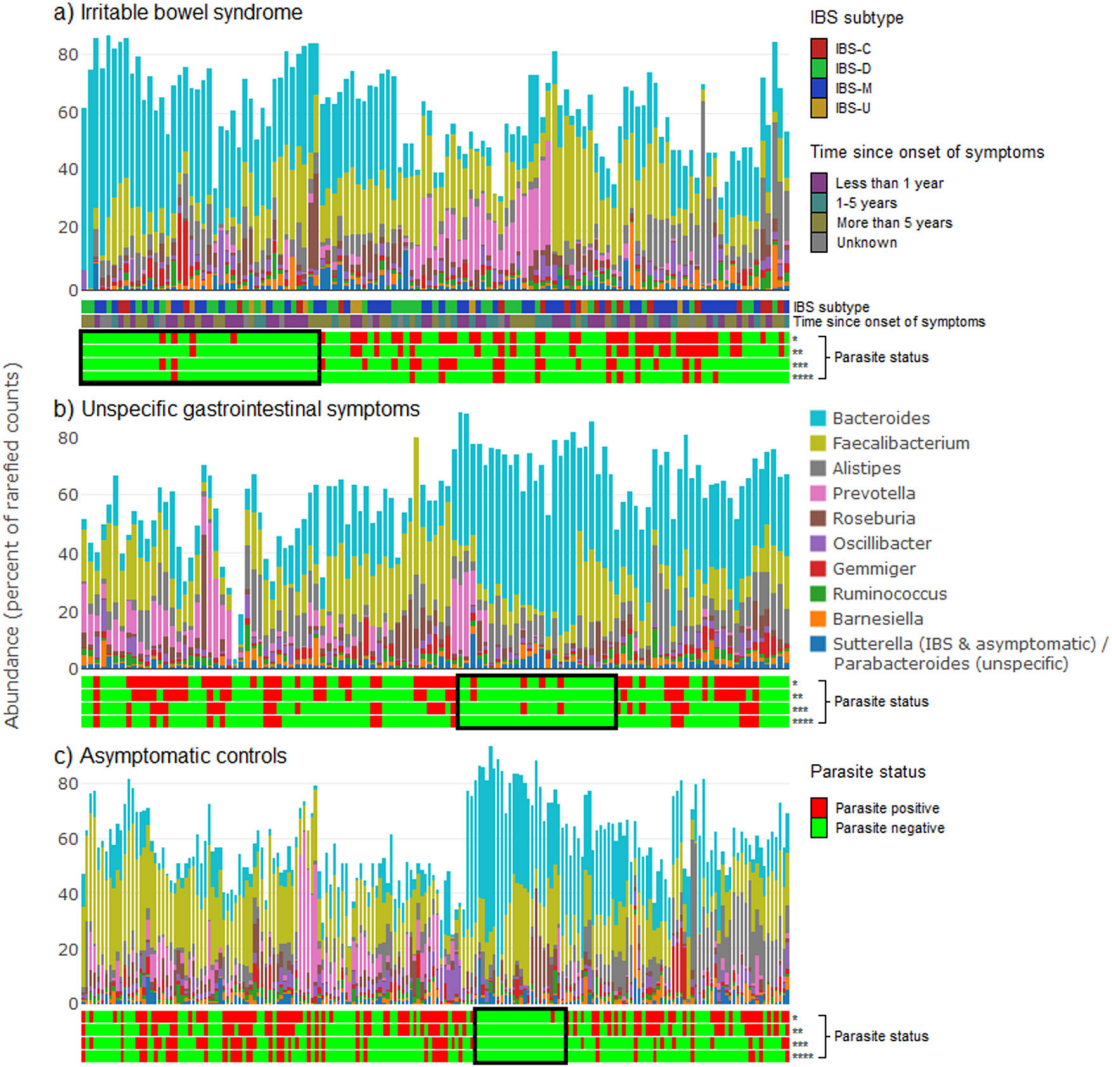
Relative abundance of the ten most abundant bacterial genera found in each of the three symptom groups. Each bar illustrates the bacterial abundance in one stool sample from an individual. Samples are ordered according to a hierarchical clustering, grouping samples with a similar bacterial composition. Plot **a** includes samples from individuals with irritable bowel syndrome (IBS), plot **b** includes samples from individuals with unspecific GI symptoms, and plot **c** includes samples from asymptomatic controls. The two horizontal bars below plot **a** show the IBS subtype and time since onset of symptoms of each sample. Additionally, the four horizontal bars below each of the three plots indicate the presence or absence of *, any parasite; **, Dientamoeba fragilis; ***, Blastocystis; and ****, multiple parasites. Notable clusters of samples with low prevalence of parasites are highlighted with a black box

The abundance of the ten most common bacterial genera in combination with parasite colonization status is visualized in Fig. 3. In general, a low frequency of parasites was found in samples with a high proportion of *Bacteroides*. The samples with a high proportion of *Bacteroides* had more than 50% of the bacterial abundance accounted for by the ten most common genera. Samples not dominated by *Bacteroides* consisted to a greater extent of several different, low-abundant genera, reflecting a more diverse microbiome.

There was no clear clustering of IBS subtype or time since onset of symptoms in relation to the clustering based on abundance of genera (Fig. 3).

### Prevalence of bacterial species

Based on our definition of species of particular relevance to IBS, five species were found at a higher prevalence in the IBS group with the difference in prevalence reaching statistical significance for all five (Table 4). All five species, except *Blautia faecis*, were also found at a higher prevalence in parasite-negative samples compared with parasite-positive samples (Table 4).

**Table 4:** Bacterial taxa classified at species level with a difference in prevalence of more than 10% between the IBS group and the group of asymptomatic controls

	IBS *n* = 119	Asymptomatic *n* = 186	Unspecific GI symptoms *n* = 114	Any parasite Positive *n* = 197 Negative, *n* = 222	*D. fragilis* Positive *n* = 134 Negative *n* = 285	*Blastocystis* Positive *n* = 100 Negative *n* = 319	Multiple parasites Positive *n* = 67 Negative *n* = 352
Species most prevalent in IBS							
* Alistipes finegoldii*	**70 (59)** ^a^	83 (45)	58 (51)	Positive: 86 (44)	Positive: 63 (47)	Positive: 36 (36)	Positive: 23 (34)
				**Negative: 125 (56)** ^b^	Negative: 148 (52)	**Negative: 175 (55)** ^**b**^	**Negative: 188 (53)** ^b^
* Bacteroides fragilis*	**53 (45)** ^a^	61 (33)	47 (41)	Positive: 60 (30)	Positive: 43 (32)	Positive: 30 (30)	Positive: 19 (28)
				**Negative: 101 (45)** ^b^	**Negative: 118 (42)**	**Negative: 131 (41)** ^b^	**Negative: 142 (40)**
* Blautia faecis*	**102 (86)** ^a^	134 (72)	88 (77)	Positive: 156 (79)	Positive: 107 (80)	Positive: 77 (77)	Positive: 51 (76)
				Negative: 168 (76)	Negative: 217 (76)	Negative: 245 (77)	Negative: 273 (76)
* Flavonifractor plautii*	**52 (44)** ^a^	44 (24)	31 (27)	Positive: 29 (14)	Positive: 107 (8)	Positive: 9 (9)	Positive:11 (14)
				**Negative: 98 (46)** ^b^	**Negative: 114 (40)** ^b^	**Negative: 118 (37)** ^b^	**Negative: 115 (33)** ^b^
* Parabacteroides distasonis*	**73 (61)** ^a^	78 (42)	66 (58)	Positive: 86 (44)	Positive: 51 (38)	Positive: 47 (47)	Positive: 24 (34)
				**Negative: 131 (59)** ^b^	**Negative: 165 (58)** ^b^	Negative: 169 (53)	**Negative: 193 (55)** ^b^
Species most prevalent in asymptomatic							
* Bifidobacterium adolescentis*	68 (57)	**126 (68)**	80 (70)	**Positive: 143 (73)** ^b^	**Positive: 99 (74)** ^b^	**Positive: 74 (74)** ^b^	**Positive: 50 (75)**
				Negative: 131 (59)	Negative: 177 (62)	Negative: 200 (63)	Negative: 224 (64)
* Clostridium bartlettii*	23 (19)	**56 (30)** ^a^	54 (47)	Positive: 66 (34)	Positive: 50 (37)	Positive: 27 (27)	Positive: 18 (27)
				Negative: 66 (28)	Negative: 83 (29)	Negative: 105 (33)	Negative: 114 (32)
* Coprococcus eutactus*	48 (40)	**92 (50)**	59 (52)	**Positive: 122 (62)** ^b^	**Positive: 88 (66)** ^b^	**Positive: 63 (63)** ^b^	**Positive: 45 (67)** ^b^
				Negative: 77 (35)	Negative: 111 (39)	Negative: 137 (43)	Negative: 154 (44)
* Dialister invisus*	28 (24)	**63 (34)** ^a^	41 (36)	Positive: 58 (29)	Positive: 44 (33)	Positive: 22 (22)	Positive: 12 (18)
				Negative: 74 (33)	Negative: 88 (31)	**Negative: 112 (35)** ^b^	**Negative: 120 (34)** ^b^
* Roseburia faecis*	95 (80)	**167 (90)** ^a^	100 (88)	Positive: 176 (89)	Positive: 121 (90)	Positive: 90 (90)	Positive: 60 (90)
				Negative: 186 (84)	Negative: 242 (85)	Negative: 274 (86)	Negative: 302 (86)

Species prevalence is reported according to symptom group and parasite colonization status *GI* gastrointestinal, *IBS* irritable bowel syndrome

^a^Significant difference in prevalence of species between the IBS group and asymptomatic controls by *χ*^2^-test

^b^Significant difference in prevalence of species between samples positive and negative for parasites according to parasite colonization status by *χ*^2^-test

Five species were found at a higher prevalence in asymptomatic controls compared with the IBS group, reaching statistical significance for three (Table 4).

For the group with unspecific GI symptoms, 21 species were found with a difference in prevalence of at least 10% compared with the IBS group and/or the asymptomatic controls (Table S2). In relation to parasite colonization status, 34 species were found with a difference in prevalence of at least 10% between samples positive and negative for parasites (Table S3).

## Discussion

In this study, we investigated the composition of the bacterial microbiome in IBS in relation to colonization by common intestinal parasites. The bacterial microbiome displayed marked differences in relation to parasite colonization status, and we found indications of an altered relationship between parasites and the bacterial microbiome in individuals with IBS.

In general, little difference in the bacterial microbiome between the IBS group and asymptomatic controls was observed. A higher abundance of *Bacteroides* and a lower abundance of *Faecalibacterium* were found in individuals with IBS. However, there was no difference in the overall composition of the microbiome between IBS and asymptomatic controls according to richness and diversity or by PCoA.

On the other hand, clear differences in the structure of the bacterial microbiome according to parasite colonization status were observed in several analyses. PCoA plots revealed a more distinct dissimilarity between samples in relation to parasite colonization status than in relation to symptom group. Individuals colonized with parasites exhibited a more diverse and rich bacterial microbiome compared with individuals not colonized by parasites, which confirms earlier findings related to *Blastocystis*^36,37^.

High bacterial diversity and richness is considered an attribute of a healthy microbiome^38^, and a decline in diversity and richness has been linked to several disease states^39–41^. The concept of parasites as inhabitants of a healthy, diverse microbiome complements associations of parasites to health-promoting features of the microbiome^42,43^ and the hygiene hypothesis^44–46^.

Higher richness and diversity in parasite-positive samples was observed in asymptomatic individuals and in individuals with unspecific GI symptoms but could not be generalized to the IBS group, which suggests that the relationship between parasites and the bacterial microbiome is altered in individuals with IBS compared with non-IBS individuals.

The overall association between abundance of bacteria and parasite colonization appeared similar in the three symptom groups (Fig. 3); samples with a high proportion of *Bacteroides* were dominated by this genus to an extent leading to less diversity, also reflected by a very low frequency of colonization with parasites.

The inverse relationship between colonization with *D. fragilis* and *Blastocystis* and a high abundance of *Bacteroides* confirms earlier findings^20,37,47^.

Interestingly, even though we did not find any major differences in the composition of the microbiome between IBS and controls, the bacterial microbiome of individuals belonging to the group with unspecific GI symptoms displayed distinct features.

The symptoms represented by the group with unspecific symptoms are very common, as they are reported by 29.5% of the Danish background population^48^. Most individuals with unspecific symptoms fulfilled ≥two of three IBS criteria regarding the association of abdominal pain to bowel habits as defined by the Rome III criteria, but symptoms were reported to occur at a lower frequency and of shorter duration (<6 months)^48^. Our data suggest that these unspecific GI symptoms are associated with a more pronounced alteration in the bacterial microbiome compared with the chronic condition of IBS. The group with unspecific GI symptoms reported a high yearly incidence of IBS (18.7%)^21^, and investigating a possible association between the high incidence of IBS and the detected aberrations of the bacterial microbiome in this group, would be of great interest. Interestingly, the finding of pronounced changes of the bacterial microbiome in the group with unspecific symptoms rather than in the IBS group does not apply to findings related to parasite colonization. Individuals with IBS displayed the greatest deviation from the high prevalence of parasite colonization observed in asymptomatic controls.

Findings of a similar diversity and richness in individuals with IBS and controls confirms a previous study^2^, while most studies found a lower diversity in the IBS population^3–5,7,8,12–14,49,50^, and two studies reported a higher diversity in IBS^15,51^. The finding of *Bacteroidetes* being more prevalent in IBS is supported by some studies^3,5,15^ while others find that *Bacteroidetes* is more abundant in healthy controls^8,11–13^.

When comparing the ten species with a difference in prevalence between IBS and controls (Table 4) with data from previous studies^2–16^, we could not find support for any particular clinical relevance to IBS.

The strengths of our study include the fact that stool samples were collected in the background population and that we had large sets of samples representing each symptom group. We compared the structure of the microbiome in individuals with IBS not only with that of asymptomatic controls, but also with individuals with unspecific GI symptoms, as alterations in the microbiome should not only enable discrimination between IBS and asymptomatic individuals, but also between individuals with IBS and those with GI symptoms not categorized as IBS, in order to be of clinical relevance. We established the presence of parasites by both sequencing of the 18s gene and by routine laboratory tests and a low sensitivity for *D. fragilis* and to some extent *Entamoeba* spp. was evident by 18S sequencing. This is a relevant finding with regard to interpretation of analyses based on sequencing data as they could underestimate the presence of *D. fragilis* in samples.

Limitations to the present study include the limited characterization of the study population. Information on demographics and characterization of GI symptoms according to the Rome III criteria were available, but we had no information on possible diagnoses not related to the GI tract, medication use and diet, which are all factors that could influence the composition of the gut microbiome. Stool samples were returned by postal mail in tubes with no additives, which could affect the composition of the microbiome compared with immediate freezing at −80 °C^52^. However, studies have shown that the effect of interperson variability is larger than effect of storage methods on bacterial composition^53,54^.

Numerous studies of the bacterial microbiome in IBS have not been able to identify distinct reproducible changes in relation to IBS which questions a possible significant and causal relation. However, many factors have the potential to affect data on the microbiome; both factors unrelated to the study setting and factors embedded in the study design leading to variance in findings between studies. Even so, a distinct feature of the bacterial gut microbiome directly involved in the pathogenesis of IBS should be expected to be evident with some coherence between studies. Two studies have found that only a subgroup of IBS patients differ in composition of the bacterial microbiome compared with healthy controls^8,15^, which could explain why no distinct changes were found in the overall comparison of IBS and controls. Data from this study did not cluster IBS samples into such subgroups (data not shown).

This study sought to analyze the bacterial microbiome in the context of parasite colonization status and illustrated the importance of integrating the various taxonomic kingdoms when studying the composition of the microbiome. Observations of parasite colonization being associated to healthy features of the gut microbiome should differentiate our view of intestinal parasites beyond the focus on pathogenicity. Further investigation into the altered relationship of parasites and the bacterial microbiome in IBS could contribute to clarifying its potential for distinguishing IBS from both asymptomatic individuals and individuals with GI symptoms not fulfilling the criteria for IBS.

In conclusion, little difference in bacterial composition was observed between individuals with IBS and healthy controls. Parasite colonization was associated to a healthy state, with parasites colonizing 50% of asymptomatic controls, while a significantly lower colonization rate was observed in individuals with IBS. The bacterial microbiome displayed clear differences related to parasite colonization status. Presence of parasites was associated to a rich and diverse microbiome in healthy controls and individuals with unspecific GI symptoms; however, this association was altered in individuals with IBS. The significance of this should be investigated further to understand possible trans-kingdom changes of the microbiome in IBS.

## Study Highlights

### What is current knowledge?

- Alterations of the bacterial microbiome is reported in IBS, although with inconsistent findings.
- Individuals with IBS are less frequently colonized with intestinal parasites.

### What is new here?

- Colonization with parasites is associated with a high diversity and richness of the bacterial microbiome in asymptomatic controls and individuals with non-IBS symptoms.
- This association between parasites and bacteria is altered in individuals with IBS.

## Electronic supplementary material

Supplementary material
