# Supplementary material for: Characteristics of the bacterial microbiome in association with common intestinal parasites in irritable bowel syndrome
Source: Clin Transl Gastroenterol. 2018 Jun 19;9(6):161. doi: 10.1038/s41424-018-0027-2 (PMC6006308; doi:10.1038/s41424-018-0027-2)
Supplement: Supplementary file 1 — Supplementary material [file 41424_2018_27_MOESM1_ESM.docx]

**Supplementary material:**

**DNA amplification primers:** We targeted the 18S ribosomal (r) RNA gene to ensure amplification of as broad a spectrum of eukaryotic organisms as possible using as few primers as possible. Three different primer sets were used; G3F1/G3R1 (5’-GCCAGCAGCCGCGGTAATTC-3’ / 5’-ACATTCTTGGCAAATGCTTTCGCAG-3’), G4F3/G4R3 (5’-CAGCCGCGGTAATTCCAGCTC-3’ / 5’-GGTGGTGCCCTTCCGTCAAT-3’), and G6F1/G6R1 (5’-TGGAGGGCAAGTCTGGTGCC-3’ / 5’-ACGGTATCTGATCGTCTTCGATCCC-3’). The G3-1 and G6-1 primers target the hyper-variable regions V3–V4 of the 18S rRNA gene, while the G4-3 primers target V3–V5. For prokaryotes, we targeted the 16S rRNA gene, using a modified version of the published universal prokaryotic primers 341F/806R, amplifying the V3–V4 hyper-variable region (1). The forward primer had three additional nucleotides attached in the 5’ end (5’-ACTCCTAYGGGRBGCASCAG-3’, 341F3), and the reverse primer had five additional nucleotides attached to the 5’ end (5’-AGCGTGGACTACNNGGGTATCTAAT-3’, 806R5).

Due to sequence variation across the selected primer regions, primer sequences were selected based on the highest number of species to be targeted. However, certain species do not have a perfect match to the selected primers, and thus have a lower amplification sensitivity. In-house testing of the utilized primers showed bias against certain parasites, including but not limited to *D. fragilis* and *Entamoeba*, which have 2-3 mismatches to the primers, especially in the presence of *Blastocystis* and fungi, which have a perfect match.

**Library prep**: Purified genomic DNA from each sample was initially amplified using the 16S and 18S primers. The 18S rDNA was amplified using initial denaturation at 95 °C for 3 min, 20 cycles of 95 °C for 1 min, 60 °C for 1 min, and 72 °C for 30 sec, and final elongation at 72°C for 4 min. The 16S rDNA was amplified using initial denaturation at 95 °C for 2 min, 20 cycles of 95 °C for 30 sec, 60 °C for 1 min, and 72 °C for 30 sec, and final elongation at 72 °C for 7 min. The products from PCR1 were prepared for sequencing by a second PCR, attaching adaptor A, index i5, and a forward sequencing primer site to the 5’-end of the amplicons, and adaptor B, index i7, and a reverse sequencing primer site to the 3’-end of the amplicons. Hence, four different PCR amplicon products were generated for each sample. DNA was quantified using the Quant-IT^TM^ dsDNA High Sensitive Assay Kit (Thermo Fisher Scientific, Waltham, MA, USA), and PCR2 products were pooled in equimolar amounts between samples. Amplicons less than 300 base pairs (bp) and larger than 1000 bp were removed from the pooled amplicon library (PAL) by Agencourt AMPure XP bead (Beckman Coulter, Brea, CA, USA) purification in a two-step process. Initially, small DNA fragments shorter than 300 bp were removed using a 10:24 µL PAL:AMPure beads ratio, following the manufacturer’s protocol. Second, DNA fragments longer than 1000 bp were removed by a 10:16 µL purified PAL:AMPure beads ratio, using the same approach. The resulting AMPure beads purified PAL was denoted bPAL. The bPAL was diluted to its final concentration of 11.5 pM DNA with a 0.001 N NaOH concentration, used for sequencing on the Illumina MiSeq desktop sequencer (Illumina Inc., San Diego, CA 29122, USA). The library was sequenced with the 500-cycle MiSeq Reagent Kit V2 in a 2 x 250 bp setup (Illumina Inc., San Diego, CA 29122, USA).

**Mapping raw sequence data to species**

Mapping of raw sequence data to unique taxonomic groups was performed using BION, a k-mer based mapping software. The MiSeq software initially demultiplexed raw sequence data into sample-specific forward- and reverse-reads Fastq files, and sequences originating from each of the four amplifications were extracted. Raw sequences were then cleaned, trimmed, and if possible, joined into single sequences of high-quality nucleotides. Raw sequences were trimmed using a minimum Phred quality score of 20, for at least 14 of 15 bp in both the 3’- and 5’-end of the forward read and 28 out of 30 bp for reverse reads. Read pairs were joined, if there were overlaps of at least 18 bp with at least 90% similarity, and if sequences did not overlap, the forward and reverse reads were put end-to-end and the breakpoint was noted. The cleaned sequences were collapsed for 100% identical sequences in order to reduce the number of sequences. Following collapse, the sequences were clustered based on 99% identity for chimera check, and non-chimeric sequences of minimum 300 bp proceeded to mapping. Sequences were then broken up into lists of 8-mers, skipping single- or stretches of low quality-nucleotides, and the breakpoint of non-overlapping paired-end reads. 8-mers were generated using a size step of 4 bp. Reference sequences were broken down into 8-mers, with a list of unique 8-mers representing each reference sequence. Similarities between query sequences and reference sequences were calculated based on the number of 8-mers, which were 100% identical between the query sequence and the reference sequence. In order to increase specificity and reduce noise based on random similarities between 8-mers within and outside the amplified area, sequences were only compared against the 340–807 region in the RDP 11.04 reference database (2) for sequences amplified by the 16S primers, and the corresponding region in the SILVA version 123 reference database (3) for sequences amplified by the 18S primers. With regard to the use of SILVA, an improved in-house seven-tier taxonomy was applied for eukaryotes, similar to the tiers defined for prokaryotes. Tables were developed for phylum, class, order, family, genus, species and sequence levels.

**Filtration**

Sequence data was filtered by applying a threshold of 20 sequences for each unique taxon to be considered present in a sample. The threshold was based on in-house observations of the current sequencing setup, indicating a demultiplexing error rate of approximately 0.1 % of the sequence counts of the most abundant prokaryotes (results not shown). As the highest count observed was 20,000, the applied cut-off for positive scoring of any pro- and eukaryotic species was 20. The prokaryotic primers had little or no notable biases towards/against any specific species; meanwhile, with regard to the eukaryotic primers, in-house observations indicated a strong bias towards *Blastocystis* sp*.*, resulting in increased sensitivity for detection and potential contaminations. Earlier in-house studies had indicated ~1.6 % of the sequence counts of the most abundant positive sample should be used as cut-off for positive identification of *Blastocystis* sp. (results not shown). With *Blastocystis* sp. counts amounting to 105,000 sequences, application of a bias of 1.6 % resulted in a cut-off of 1,700 for *Blastocystis* sp.

Taxa present in less than three samples were also removed in the filtration.

Sequencing data on parasites were manually interpreted, aiming to identify and removing potential artifacts.

**Statistics**

For bacterial species present with a difference in prevalence of at least 10% between the IBS group and asymptomatic controls, the mean number of sequences mapped to each species was compared between symptom groups and between parasite-positive and -negative samples.

**Results**

Comparison of the mean number of sequences mapped to the specific species between symptom groups and between parasite-positive and -negative samples showed a difference of less than 100 sequences between the groups (data not shown).

References

1. Yu Y, Lee C, Kim J, et al. Group-specific primer and probe sets to detect methanogenic communities using quantitative real-time polymerase chain reaction. Biotechnol Bioeng. 2005;89:670–679.
2. Cole, J. R., Q. Wang, J. A. Fish, et al. 2014. Ribosomal Database Project: data and tools for high throughput rRNA analysis Nucl Acids Res 42(Database issue):D633-D642.
3. Pruesse E, Quast C, Knittel K, et al. SILVA: a comprehensive online resource for quality checked and aligned ribosomal RNA sequence data compatible with ARB. [Nucl Acids Res 2007;35:7188-96](http://nar.oxfordjournals.org/content/35/21/7188.full).

Table S1. Parasites detected by sequencing and routine laboratory methods in all samples (n=419). Conventional methods included microscopy, short-term *in vitro* culture for *Blastocystis*, and qPCR.

|  | Positive by sequencing and routine laboratory methods | Positive by sequencing only | Positive by routine laboratory methods only | Negative by both sequencing and routine laboratory methods | Total no. of positive samples |
| --- | --- | --- | --- | --- | --- |
| *Endolimax (nana)* | 2 | 3 | 4 | 410 | 9 |
| *Blastocystis* | 70 | 20 | 10 | 319 | 100 |
| *Dientamoeba (fragilis)* | 14 | 0 | 120 | 285 | 134 |
| *Entamoeba (coli, dispar, hartmanni, histolytica)** | 8 | 4 | 20 | 387 | 32 |
| *Giardia (intestinalis)* | 0 | 0 | 2 | 417 | 2 |
| *Vermamoeba* | 0 | 1 | 0 | 418 | 1 |

*By sequencing E. dispar was detected in samples from 2 individuals and E. coli was detected in samples from 10 individuals.

Table S2. Prokaryotic taxa classified at species level with a difference in prevalence of at least 10% between individuals with unspecific GI symptoms and individuals with IBS and/or asymptomatic controls. Prevalence according to symptom group and parasite colonization status is reported for each given species.

Prevalence

|  |  |  |  |  |  |  | |  |
| --- | --- | --- | --- | --- | --- | --- | --- | --- |
|  | IBS,  n=119 | Asymptomatic, n=186 | Symptom group, **n (%)**  Unspecific GI symptoms, n=114 | Any parasite, Positive n=197  Negative n=222 | Parasite colonisation status, **n (%)**  *Dientamoeba fragilis*  Positive n=134  Negative n=285 | | *Blastocystis*  Positive n=100  Negative n=319 | Multiple parasites  Positive n=67  Negative n=352 |
| *Akkermansia muciniphila* | 47 (40) | 75 (40) | **66 (58)**^a^ | **Positive: 99 (50)^b^**  Negative: 89 (40) | **Positive: 77 (58)^b^**  Negative: 111 (39) | | Positive: 47 (47)  Negative: 141 (44) | **Positive: 36 (54)**  Negative: 152 (43) |
| *Alistipes senegalensis* | 15 (13) | 27 (15) | **30 (26)**^a^ | **Positive: 47 (24**)^b^  Negative: 25 (11) | Positive: 25 (19)  Negative: 47 (17) | | **Positive: 36 (36)^b^** Negative: 36 (11) | **Positive: 24 (36) ^b^**  Negative: 48 (14) |
| *Anaerostipes hadrus* | 91 (77) | 151 (81) | **99 (87)** | Positive: 167 (85)  Negative: 173 (78) | Positive: 115 (86)  Negative: 225 (79) | | Positive: 80 (80)  Negative: 260 (82) | Positive: 54 (81)  Negative: 286 (81) |
| *Bacteroides intestinalis* | 9 (8) | 21 (11) | **20 (18)** | Positive: 24 (12)  Negative: 26 (12) | Positive: 12 (9)  Negative: 38 (13) | | Positive: 14 (14)  Negative: 36 (11) | Positive: 7 (10)  Negative: 43 (12) |
| *Bacteroides caccae* | 62 (52) | 83 (45) | **65 (57)** | Positive: 101 (51)  Negative: 109 (49) | Positive: 66 (49)  Negative: 144 (51) | | Positive: 51 (51)  Negative: 159 (50) | Positive: 31 (46)  Negative: 179 (51) |
| *Clostridium celatum* | 17 (14) | 34 (18) | **47 (41)**^a^ | Positive: 46 (23)  Negative: 51 (23) | Positive: 30 (22)  Negative: 67 (24) | | Positive: 18 (18)  Negative: 79 (25) | Positive: 10 (15)  **Negative: 87 (25)** |
| *Clostridium ruminatum* | 1(1) | 2 (1) | **12 (11)**^a^ | Positive: 7 (4)  Negative: 8 (4) | Positive: 5 (4)  Negative: 10 (4) | | Positive: 2 (2)  Negative: 13 (4) | Positive: 0  Negative: 15 (4) |
| *Collinsella aerofaciens* | 81 (68) | 134 (72) | **89 (78)** | Positive: 147 (75)  Negative: 157 (71) | Positive: 92 (67)  Negative: 212 (75) | | Positive: 78 (78)  Negative: 226 (71) | Positive: 50 (75)  Negative: 254 (72) |
| *Coprococcus comes* | 82 (69) | 134 (72) | **92 (81)** | Positive: 151 (77)  Negative: 157 (71) | Positive: 98 (73)  Negative: 210 (74) | | **Positive: 86 (86)^b^**  Negative: 222 (70) | **Positive: 56 (84)^b^**  Negative: 252 (72) |
| *Desulfovibrio piger* | 15 (13) | 20 (11) | **25 (22)**^a^ | Positive: 35 (18)  Negative: 24 (11) | Positive: 22 (16)  Negative: 37 (13) | | Positive: 19 (19)  Negative: 40 (13) | Positive: 14 (21)  Negative: 45 (13) |
| *Gordonibacter pamelaeae* | 4 (3) | 10 (5) | **19 (17)**^a^ | Positive: 14 (7)  Negative: 19 (9) | Positive: 11 (8)  Negative: 22 (8) | | Positive: 5 (5)  Negative: 28 (9) | Positive: 3 (4)  Negative: 30 (9) |
| *Haemophilus parainfluenzae* | 32 (27) | 45 (24) | **43 (38)**^a^ | Positive: 64 (32)  Negative: 56 (25) | Positive: 44 (33)  Negative: 76 (27) | | Positive: 34 (34)  Negative: 86 (27) | Positive: 19 (28)  Negative: 101 (29) |
| *Methanobrevibactor smithii* | 12 (10) | 23 (12) | **30 (26)**^a^ | **Positive: 48 (24)^b^**  Negative: 17 (8) | **Positive: 36 (27)^b^**  Negative: 29 (10) | | **Positive: 27 (27)^b^** Negative: 38 (12) | **Positive: 21 (31)^b^**  Negative: 44 (13) |
| *Oxalobacter formigenes* | 18 (15) | 28 (15) | **29 (25)**^a^ | **Positive: 48 (24)^b^**  Negative: 27 (12) | Positive: 30 (22)  Negative: 45 (16) | | **Positive: 28 (28)^b^**  Negative: 47 (15) | **Positive: 20 (30)^b^**  Negative: 55 (16) |
| *Roseburia intestinalis* | 41 (35) | 59 (32) | **56 (49)**^a^ | Positive: 69 (35)  Negative: 86 (39) | Positive: 46 (34)  Negative: 109 (38) | | Positive: 34 (34)  Negative: 121 (38) | Positive: 19 (28)  **Negative: 136 (39)** |
| *Roseburia hominis* | **53 (45)** | **87 (47)** | 39 (34)^a^ | Positive: 86 (44)  Negative: 93 (42) | Positive: 62 (46)  Negative: 117 (41) | | Positive: 48 (48)  Negative: 131 (41) | **Positive: 35 (52)**  Negative: 144 (41) |
| *Ruminococcus callidus* | 47 (40) | 71 (38) | **55 (48)** | Positive: 79 (40)  Negative: 94 (42) | Positive: 51 (38)  Negative: 122 (43) | | Positive: 35 (35)  Negative: 138 (43) | Positive: 20 (30)  Negative: 153 (44) |
| *Ruminococcus bromii* | 66 (56) | 116 (62) | **81 (71)**^a^ | **Positive: 136 (69)^b^**  Negative: 127 (57) | **Positive: 95 (71)^b^**  Negative: 168 (59) | | Positive: 65 (65)  Negative: 198 (62) | Positive: 40 (60)  Negative: 223 (63) |
| *Slackia isoflavoniconvertens* | 21 (18) | 21 (11) | **28 (26)**^a^ | Positive: 37 (19)  Negative: 33 (15) | Positive: 20 (15)  Negative: 50 (18) | | Positive: 20 (20)  Negative: 50 (16) | Positive: 13 (19)  Negative: 57 (16) |
| *Suterella wadsworthensis* | **61 (51)** | **104 (56)** | 52 (46) | Positive: 105 (53)  Negative: 112 (50) | Positive: 72 (54)  Negative: 45 (51) | | Positive: 54 (54)  Negative: 163 (51) | Positive: 36 (54)  Negative: 181 (51) |
| *Veilonella dispar* | 3 (3) | 7 (4) | **20 (18)** | Positive: 14 (7)  Negative: 16 (7) | Positive: 8 (6)  Negative: 22 (8) | | Positive: 7 (7)  Negative: 23 (7) | Positive: 3 (4)  Negative: 27 (8) |

GI, gastrointestinal; IBS, irritable bowel syndrome. ^a^: Significant difference between all three symptom groups by χ^2^test. ^b^: Significant difference between positive and negative samples by χ^2^ test.

Table S3. Prevalence of bacterial species according to parasite colonization status. Prokaryotic taxa classified at species level with a difference in prevalence of at least 10% between parasite-positive and -negative samples in at least one of the four parasite colonization status categories. Prevalence of the given species is reported according to parasite colonization status. A prevalence at least 10% higher or lower in parasite-positive samples is indicated by green or red background color, respectively.

|  |  |  | Prevalence of bacterial species according to parasite colonization status % |  |  |  |  |  |
| --- | --- | --- | --- | --- | --- | --- | --- | --- |
|  | Parasite-positive,  n = 197 | Parasite-negative,  n = 222 | *Dientamoeba fragilis*-positive,  n = 134 | *Dientamoeba fragilis*-negative,  n = 285 | *Blastocystis-*positive,  n = 100 | *Blastocystis*-negative,  n = 319 | Multiple parasite-positive,  n = 67 | Multiple parasite-negative  n = 352 |
| *Akkermansia muciniphila* | **50** | **40** | **57** | **39** | 47 | 44 | **54** | **43** |
| *Alistipes shahii* | **87** | **73** | **89** | **76** | 87 | 78 | **90** | **78** |
| *Alistipes senegalensis* | **24** | **11** | 19 | 17 | **36** | **11** | **36** | **14** |
| *Alistipes indistinctus* | **41** | **23** | **42** | **27** | 37 | 30 | 37 | 30 |
| *Bacteroides massiliensis* | **38** | **26** | 34 | 30 | 37 | 30 | 30 | 32 |
| *Bifidobacterium longum* | **65** | **55** | **69** | **56** | 65 | 58 | **69** | **58** |
| *Bifidobacterium adolescentis* | **73** | **59** | **74** | **62** | **74** | **63** | **75** | **64** |
| *Blautia obeum* | 56 | 50 | 51 | 54 | **62** | **50** | 58 | 52 |
| *Butyrivibrio crossotus* | **24** | **4** | **24** | **8** | **30** | **8** | **34** | **9** |
| *Catenibacterium mitsuokai* | 17 | 10 | 17 | 12 | 19 | 12 | **24** | **11** |
| *Coprococcus eutactus* | **62** | **35** | **66** | **39** | **63** | **43** | **67** | **44** |
| *Coprococcus comes* | 77 | 71 | 73 | 74 | **86** | **70** | 84 | 72 |
| *Methanobrevibacter smithii* | **24** | **8** | **27** | **10** | **27** | **12** | **31** | **13** |
| *Odoribacter splanchnicus* | **94** | **83** | 95 | 86 | **96** | **86** | **97** | **87** |
| *Oxalobacter formigenes* | **24** | **12** | 22 | 16 | **28** | **15** | **30** | **16** |
| *Paraprevotella clara* | 34 | 26 | **37** | **27** | 35 | 26 | 34 | 29 |
| *Phascolarctobacterium*  *Succinatutens* | 31 | 22 | 29 | 25 | **37** | **23** | **43** | **23** |
| *Prevotella copri* | **47** | **26** | **46** | **31** | **48** | **32** | **54** | **32** |
| *Roseburia hominis* | 44 | 42 | 46 | 41 | 48 | 41 | **52** | **41** |
| *Ruminococcus flavefaciens* | **13** | **3** | 11 | 6 | **18** | **4** | **18** | **6** |
| *Ruminococcus champanellensis* | 9 | 1 | 10 | 2 | **14** | **2** | **18** | **2** |
| *Ruminococcus bromii* | **69** | **57** | **71** | **59** | 65 | 62 | 58 | 63 |
| *Senegalimassilia anaerobia* | 26 | 18 | 21 | 22 | **34** | **18** | **31** | **20** |
| *Victivallis vadensis* | **24** | **9** | **25** | **13** | **28** | **13** | **30** | **14** |
| *Alistipes finegoldii* | **44** | **56** | 47 | 52 | **36** | **55** | **34** | **53** |
| *Bacteroides fragilis* | **30** | **45** | **32** | **42** | **30** | **41** | **28** | **40** |
| *Bifidobacterium bifidum* | 16 | 19 | 20 | 17 | **10** | **20** | 12 | 19 |
| *Bilophila wadsworthia* | 56 | 57 | 60 | 55 | **47** | **60** | 48 | 58 |
| *Clostridium leptum* | **5** | **19** | **4** | **16** | **5** | **15** | **4** | **14** |
| *Dialister invisus* | 29 | 34 | 33 | 31 | **22** | **35** | **18** | **34** |
| *Flavonifractor plautii* | **12** | **46** | **8** | **40** | **9** | **37** | **9** | **34** |
| *Parabacteroides distasonis* | **44** | **59** | **38** | **58** | 47 | 53 | **36** | **55** |
| *Phascolarctobacterium*  *faecium* | 19 | 23 | 18 | 22 | 15 | 23 | **10** | **23** |
| *Ruminococcus torques* | **17** | **34** | **15** | **31** | **16** | **29** | **10** | **29** |

Figure S1. A principal coordinate analysis visualizing differences in the bacterial microbiome between samples from individuals with IBS, individuals with unspecific GI symptoms and asymptomatic controls. All samples are included in analysis (n=419) and dots are colored according to the three symptom groups. The closer the dots are ordinated, the more similar. Axes summarize the variability in the data and the value indicates the variation captured in the axis.

gjhhj


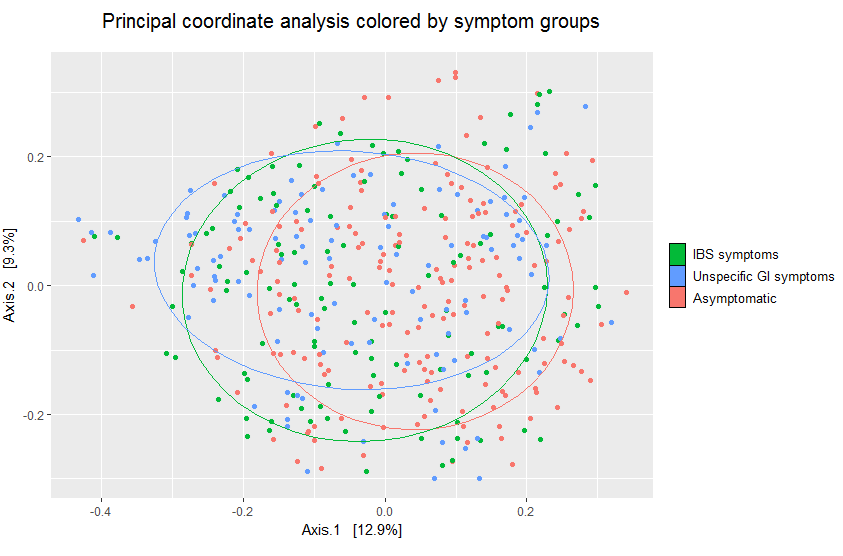


GI: Gastro intestinal; IBS: Irritable bowel syndrome
